# Foreign-body Ingestions in Children During COVID-19 Pandemic in a Pediatric Referral Center

**DOI:** 10.1097/PG9.0000000000000018

**Published:** 2020-11-11

**Authors:** Antonio Pizzol, Caterina Rigazio, Pier Luigi Calvo, Federico Scottoni, Alessandro Pane, Fabrizio Gennari, Fabio Cisarò

**Affiliations:** From the * Pediatric Gastroenterology Unit, Regina Margherita Children’s Hospital, Azienda Ospedaliera-Universitaria Città della Salute e della Scienza di Torino, Turin, Italy; † Department of Pediatric General Surgery, Regina Margherita Children’s Hospital, Azienda Ospedaliera-Universitaria Città della Salute e della Scienza di Torino, Turin, Italy.

**Keywords:** COVID-19, foreign-body ingestion, button batteries

## Abstract

In February 2020, the COVID-19 pandemic overwhelmed Italy. We retrospectively reviewed all attendances and emergency (A&E) admissions due to foreign-body ingestions (FBIs) to an Italian pediatric referral hospital, from February 24 to April 24, 2020, COVID-19 lockdown and compared them with the same period in the previous 4 years. A total of 101 cases were recorded. Mean age of admission was 4.6 years. Groups did not differ for gender (*P* = 0.4) or age (*P* = 0.3). Among FBIs ingestions, 24.0% occurred in children with <2 years of age and 47.5% in children from 2 to 6 years of age. In the 2020 study period, 9 patients were seen for batteries ingestion compared with a median value of one among compared periods. The rates of batteries ingestions increased significantly over the observational period (*P* < 0.001). We report a dramatic increase in batteries ingestions in children, a potentially fatal event, during the COVID-19 pandemic lockdown.

**What Is Known**

- Foreign-body ingestions (FBIs) in children are a commonly reported incident.
- Nearly all of FBIs happen at home.
- Among FB, batteries represent nearly 7% of ingestions but may result in severe complications and eventually fatalities.

**What Is New**

- Exceptional scenario, as COVID-19 pandemic, abruptly change family lifestyle.
- Children forced at home for pandemic contingency have an increased risk of potentially fatal FBIs, as button batteries, compared with nonpandemic periods.

**A**s of February 20, 2020, the incidence of COVID-19 in Italy began to rapidly escalate; on March 9, a national lockdown was announced by the Italian Prime Minister. A total of 3.6 million workers were forced to stay at home, and it has been estimated that nearly 11 million children were left home from school ^(1)^, resulting in severe burden for families in the management of their children.

By April 25, 2020, Italy had the second highest number of COVID-19 infections and the greatest number of deaths in Europe ^(2)^.

At the Regina Margherita Children’s Hospital in Turin, Northern Italy, the on-call endoscopic team performed a relatively high number of procedures for foreign-body removal during the lockdown period, while the routine procedures plummeted. We performed a retrospective analysis of the overall admission rate to the emergency department for foreign-body ingestions (FBIs) during the lockdown period and compared it with the exact same 2-months period in each of the previous 4 years. Aim of the study is to evaluate whether the extreme/forced change in lifestyle imposed by the pandemic had an impact on FB ingestions, based on the knowledge that most FBIs in children occur at home ^(3)^.

## MATERIALS AND METHODS

We retrospectively reviewed all A&E admissions due to FBIs to Regina Margherita Children Hospital A&E from February 24, 2020, first day of school closure, to April 24, 2020, and compared them with retrospective data for exactly the same 2-months period in the years 2019, 2018, 2017, and 2016, respectively. Data were retrieved from the hospital electronic medical record system (TrakCare® Electronic Medical Record System-InterSystems). This study was approved by the hospital institutional review board.

### Inclusion Criteria

All children aged less than 14 years who presented to the A&E department in the set periods with a history of witnessed or highly suspected FBI were included in the study.

FBIs were classified into 6 main categories: (1) coins (all types of coins); (2) batteries (button or disc batteries, AA, AAA); (3) jewelry (including rings and earrings); (4) food (including food impaction, large food pieces, fruit stones); (5) sharp objects (including screws, plastic frames, needles); (6) toys (wooden made, small pieces, bricks, and magnets and rocks).

### Statistical Analysis

Descriptive statistics were derived for the samples, including frequencies, percentages, maximum and minimum, averages, and medians. Fisher’s exact/χ^2^ test was utilized for continuous variables and categorical variables, respectively. Statistical analysis was performed with SPSS® v. 20. A *P* value of <0.05 was taken as significant.

## RESULTS

**FIGURE 1.**
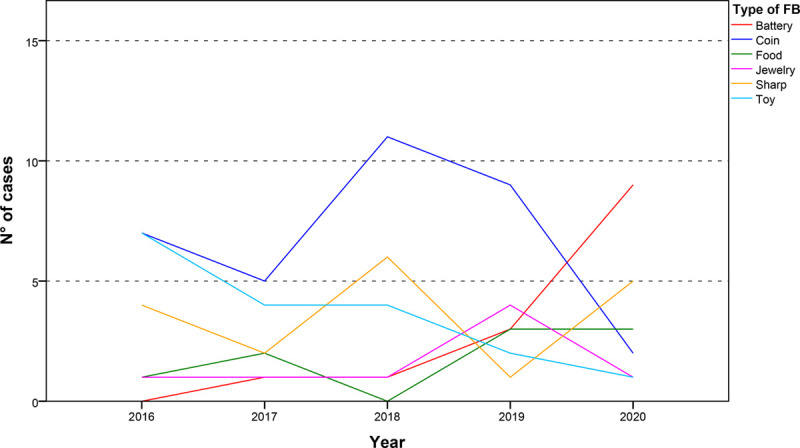
Number of FBIs by children between the 24 of February and the 24 of April in 2016, 2017, 2018, 2019, and 2020. FBIs = foreign-body ingestions.

A total of 101 cases were recorded (Table 1). Mean age of admission was 4.6 years (min 4 months, max 13.5 years, SD 3.14). Groups did not differ for gender (*P* = 0.4) or age (*P* = 0.3). The mean rate of hospital access for FBIs in the 2-month period under scrutiny was 20.2/yr (median 21.0, SD 3.1), and mean rate of ward admission for endoscopic procedures in the 2-month period under scrutiny was 3.4/yr (median 3.0, SD 2.3), with no significant differences observed between the 5 two-months examined periods (*P* = 0.16). Among FBIs ingestions, 24.0% occurred in children with <2 years of age, 47.5% in children from 2 to 6 years of age, and the others 28.5% were between 6 and 14 years of age. In the 2020 2-month study period, 9 patients were seen for batteries ingestion compared with a median value of one among the same 2-month period in the previous 4 years. On the contrary, rates of ingestion of coins dropped from a median value of 8 in the 2016–2019 2-month observational period to an observed value of 2 in the 2020 2-month period (Fig. 1). During the 2020 lockdown, 7 (33%) patients required endoscopy, all due to button batteries ingestions, with a removal rate of 77%. Two patients had minor complications (gastric ulceration for battery contact). Frequencies of groups of FBIs observed in the 2020 2-month period statistically differed from those expected based on frequencies of the previous 4 years in the same 2-month period (*P* = 0.026). Among all FBIs, the rates of batteries ingestions increased significantly over the observational period (*P* < 0.001).

**TABLE 1.** Demographic Characteristics and Number and Type of FBIs Reported at the Regina Margherita Children Hospital, Turin from 24 February to 24 April of 2016, 2017, 2018, 2019, and 2020

Characteristics/yr	2016	2017	2018	2019	2020
Age yr, mean (SD)	5.9 (3.2)	4.1 (2.8)	3.9 (2.9)	4.5 (2.9)	4.6 (3.4)
Sex (F/M)	9/11	9/6	9/14	14/8	9/12
Discharges/admissions (N)	16/4	14/1	21/2	19/3	14/7
Endoscopic procedure (N)	4	1	2	3	7
**Type of FB (N, %**)					
Battery	0	1 (7)	1 (4.5)	3 (13.5)	9 (43)
Coin	7 (35)	5 (33)	11 (48)	9 (41)	2 (9.5)
Food	1 (5)	2 (13)	0	3 (13.5)	3 (14.2)
Jewelry	1 (5)	1 (7)	1 (4.5)	4 (18)	1 (4.5)
Sharp	4 (20)	2 (13)	6 (26)	1 (4)	5 (24)
Toy	7 (35)	4 (27)	4 (17)	2 (9)	1 (4.5)
All	20 (100)	15 (100)	23 (100)	22 (100)	21 (100)

FBIs = foreign-body ingestions

## DISCUSSION

The 2020 COVID-19 pandemic has an abruptly modified family lifestyle due to infection containment measures. An overall decrease of up to 80% in access to the pediatric A&E departments for all causes has been observed, eventually resulting in a severely delayed access or provision of care ^(4)^.

Esophageal battery ingestion is the most critical indication for emergency endoscopy in children ^(5)^, as it is a potentially fatal event ^(6)^. During the lockdown, starting from February 24, 2020, we registered a significant increase in admissions for batteries ingestions. In the 2020 2-month lockdown study period, the absolute number of batteries ingestions resulted in a ninefold increase from the median value of the same 2-month periods under scrutiny in the previous 4 years. This increase resulted to be statistically significant. Moreover, in the 2-month lockdown period, we registered nearly half of the total battery ingestions expected for the year 2020 based on those observed in the year 2019. Indeed, over the year 2019, we registered 15 episodes of battery ingestions representing 8.7% of the annual A&E admissions due to FBIs, a data in accordance with previously reported findings in literature ^(3)^.

At this rate, the expected number of batteries ingestions in the next months of school’s closure can reach high and worrying numbers on a global scale. This added up to an already worrisome trend of batteries ingestions registered in the last decades ^(7)^. Interestingly, there were no differences in the hospital access for all FBIs during the 2-month lockdown period compared with the same 2-month periods in the previous 4 years, nor in the absolute number of endoscopic procedures performed. What did change was the type of FB, with a clear shift from coins, usually the most common FB ingested in children, to batteries?

These data may reflect a change in children habits during the pandemic. The abnormally increased time spent by children at home may have resulted in an increased use of electronic devices, an increased need of battery supplies and eventually an increment in accidents. On the other hand, the relative decrease in coin ingestions may also reflect the substantial change in both shopping habits (online) and payment methods (digital) during the COVID-19 pandemic in Italy. The steady admission rate for FBIs during the study period when compared with the same 2-month period in the previous 4 years somehow stresses the concept of an absolute risk of ingestions in children and the continuous presence of the risk itself. Indeed, children are used to ingest surrounding objects despite the type of the object itself, even if FBIs major complications and fatal events are strictly related to the quality of the foreign body itself. These data, on top of others, reported issues as the increased exposure to domestic accidents and, in particular, cleaning products and disinfectants and highlight the importance of a coordinated investment to prevent domestic injuries in children when shut at home, especially during pandemics ^(8, 9)^.

In our opinion, all the increment of casualties registered during the pandemic period should be considered as indirect COVID-19-related comorbidities, thus increasing the burden of this dramatic disease.

## CONCLUSIONS

Based on our retrospective study, we report a dramatic increase in batteries ingestions in children, a potentially fatal event, during the COVID-19-related lockdown in Italy. This study has the purpose to serve as an alarm recall to all physicians, Institutions, and Societies to help families preventing household accidents in their battle against the COVID-19 pandemic.

## ACKNOWLEDGMENTS

The authors thank Drs D.D.’O. for scientific support and L.D., S.C., and D.R. for endoscopy procedures.
